# Single-Institution Experience of Larotrectinib Therapy for Patients With *NTRK* Fusion-Positive Thyroid Carcinoma

**DOI:** 10.1210/jendso/bvae158

**Published:** 2024-09-09

**Authors:** Omar Elghawy, Adam Barsouk, Alec Heidlauf, Simon Chen, Roger B Cohen, Lova Sun

**Affiliations:** Division of Hematology/Oncology, Department of Medicine, Abramson Cancer Center, University of Pennsylvania, Philadelphia, PA 19104, USA; Division of Hematology/Oncology, Department of Medicine, Abramson Cancer Center, University of Pennsylvania, Philadelphia, PA 19104, USA; Division of Hematology/Oncology, Department of Medicine, Abramson Cancer Center, University of Pennsylvania, Philadelphia, PA 19104, USA; Division of Hematology/Oncology, Department of Medicine, Abramson Cancer Center, University of Pennsylvania, Philadelphia, PA 19104, USA; Division of Hematology/Oncology, Department of Medicine, Abramson Cancer Center, University of Pennsylvania, Philadelphia, PA 19104, USA; Division of Hematology/Oncology, Department of Medicine, Abramson Cancer Center, University of Pennsylvania, Philadelphia, PA 19104, USA

**Keywords:** papillary thyroid cancer, anaplastic thyroid cancer, poorly differentiated thyroid cancer, NTRK mutation, larotrectinib

## Abstract

**Context:**

The real world efficacy and tolerabiltiy of NTRK inhibitor larotrectinib has not yet been reported in the literature although trial data has shown promising results.

**Objective:**

We report a retrospective analysis of patients with thyroid cancer harboring *NTRK* fusions who underwent treatment with larotrectinib.

**Methods:**

A single-institution, retrospective case series of patients with *NTRK* fusion-positive thyroid cancers treated with neurotrophic tyrosine receptor kinase (*NTRK)* inhibitors from January 1, 2007, to January 1, 2023, was performed. This study was conducted at a single academic tertiary referral center. Patients with confirmed *NTRK*-fusion thyroid cancer who received larotrectinib were included. Larotrectinib was administered in accordance with clinical judgment from oncology providers. The primary end point was progression-free survival (PFS).

**Results:**

Eight patients with *NTRK* fusion-positive thyroid cancer treated with larotrectinib were identified: 4 with papillary thyroid cancer (PTC) (50%), 3 with poorly differentiated thyroid cancer (PDTC) (38%), and 1 with anaplastic thyroid cancer (ATC) (12%). The median PFS (mPFS) for all patients was 24.7 months (95% CI, 11.3-38.1). mPFS in PTC was higher than PDTC (34.6 months [24.7-48.7 months] vs 17.5 [7.1-21.1 months]; *P* = .017). The median overall survival (OS) was 43.8 months (29.8-56.8 months) overall. The single patient with ATC had a PFS and OS of 23 months. Two patients remained on treatment/alive at data cutoff, with a duration of response of 33.5 months and a median follow-up of 52 months. Patients achieved 1 complete response (12%), 6 partial responses (75%), and 1 stable disease (12%).

**Conclusion:**

In this single-institution cohort of patients with *NTRK* fusion-positive thyroid cancer, *NTRK* inhibition led to an mPFS of 25 months, with survival surpassing historic benchmarks for ATC and PDTC.

Thyroid cancer accounts for 2% of all cancer diagnoses and 0.3% of all cancer deaths in the United States [1]. Papillary thyroid cancer (PTC) is the most common histology, accounting for more than 80% of thyroid cancer cases and has a 5-year survival greater than 99%, although stage IV disease portends a 74% 5-year survival [1]. In the metastatic radioactive iodine-refractory setting, PTC responds favorably to multitarget tyrosine kinase inhibitors such as lenvatinib and sorafenib. Conversely, anaplastic thyroid carcinoma (ATC) and poorly differentiated thyroid carcinoma (PDTC) account for less than 1% of thyroid cancer diagnoses but more than 30% of thyroid cancer deaths [2]. Outcomes for these latter thyroid cancers are dismal as these diseases are often refractory to standard treatments for PTC [2]. Multimodal treatment with surgical resection, adjuvant radiation, and chemotherapy has been shown to improve survival; however, local and/or distant disease progression is common and long-term survival is rare [3].

Next-generation sequencing (NGS) has enabled the identification of targetable driver genetic alterations across solid tumors, paving the way for pantumor trials of tyrosine kinase inhibitors targeted to an individual patient's molecular profile. Neurotrophic tyrosine receptor kinase (*NTRK*) gene fusions have been described in more than 25 different types of cancer [4]. Although their overall prevalence in solid tumors is less than 1%, some studies have shown that the rate of occurrence has been estimated to be as high as 26% in pediatric PTC and 10% in adult PTC [4]. *NTRK* fusions can be detected most reliably by fluorescence in situ hybridization and next-generation DNA or RNA sequencing (NGS), and immunohistochemistry has also been explored as a surrogate marker [5].

The first *NTRK-*targeted agent approved by the US Food and Drug Administration was larotrectinib in 2018, based on tissue-agnostic approval of 3 multicenter, single-arm trials, LOXO-TRK-14001 (NCT02122913), SCOUT (NCT02637687), and NAVIGATE (NCT02576431) [6, 7]. In a combined analysis of these 3 studies, the first 55 patients (5 with thyroid cancer) evaluated with unresectable or metastatic solid tumors had an overall response rate (ORR) of 75% (95% CI, 61%-85%) [8, 9]. The most recent combined analysis of these phase 1/2 basket trials during the American Society of Clinical Oncology 2024 meeting, which included 24 DTCs, 3 PDTCs, and 7 ATCs with *NTRK* fusions, showed an impressive ORR of 65% with 3 complete responses (CRs) and 17 partial responses (PRs) (79% ORR in DTC and 14% in ATC) [10]. These patients demonstrated rapid and durable responses with a median time to response of 1.9 months, median progression-free survival (PFS) of 44.0 months, and a median overall survival (mOS) not reached at 39.8-month follow-up. The VICTORIA study, a trial comparing the outcomes of 82 clinical trial patients with *NTRK*-fusion cancers treated with larotrectinib, with 82 patients receiving alternative standard-of-care therapies in clinical practice, also demonstrated that treatment with larotrectinib was associated with longer OS, PFS, duration of treatment, and time to next treatment compared to nonlarotrectinib treatment, although the number of thyroid patients included in this trial is not publicly available [11].

In 2020, entrectinib was similarly approved based on the integrated study of the Alka-372-001, SRARTRK-1, and STARTRK-2 trials, which demonstrated an ORR of 57% in 54 patients across 10 tumor types (including 5 patients with thyroid cancer), and a median duration of response (DOR) of 10 months [4, 12, 13]. A recent comparative efficacy study suggested similar safety but a higher CR rate, significantly longer median OS, and a longer DOR for larotrectinib compared to entrectinib (with 25 and 7 thyroid patients on each, respectively) [14]. Both options, however, are currently commercially available, highly efficacious, and tolerable.

While *NTRK* inhibitors have shown impressive safety and efficacy in clinical trials, only a handful of thyroid cancer cases were included in these pantumor cohorts. Experience with larger patient cohorts has not been published widely with an isolated case report of a borderline resectable patient with ATC who demonstrated a dramatic response to neoadjuvant entrectinib [15], and several case reports of responses to larotrectinib exceeding historic standards in metastatic papillary thyroid cancers both in pediatric and adult populations [16-18]. We present a case series of 8 patients with thyroid cancer of different histologies harboring *NTRK* fusions who underwent treatment with larotrectinib.

## Materials and Methods

### Cohort Selection and Baseline Characteristics

We performed a single-institution, retrospective analysis of patients with histologically confirmed thyroid cancer with *NTRK* fusion identified on NGS testing and were treated with an *NTRK* inhibitor from 2007 to 2023. Baseline demographics, including age and sex, and disease characteristics including mutational status, stage, treatment history, toxicity, and clinical outcomes were abstracted from the electronic medical record. Disease stage was determined per the American Joint Committee on Cancer, eighth edition [19].

### Study End Points and Statistical Analysis

The primary end point was PFS, measured from initiation of *NTRK*-targeted therapy until radiographic disease progression warranting a change in systemic therapy or death from any cause, whichever occurred first. The secondary end points were OS, calculated from initiation of targeted therapy until death from any cause, DOR calculated from response until progression or death, and response rate per RECIST 1.1 criteria [20]. Patients alive at last follow-up were censored. Median PFS and OS were estimated with Kaplan-Meier methodology, and groups defined by histologic subtype were compared using the log-rank test. Adverse event information was assessed using common terminology criteria for adverse events (CTCAE) v5.0) [21]. All statistical tests were conducted in IBM SPSS version 26. All tests were 2-sided, and *P* less than .05 was considered statistically significant.

## Results

Eight patients with *NTRK* fusion-positive treated with larotrectinib were included in our cohort (no patients were treated with entrectinib). Histologies were PTC (N = 4, 50%), PDTC (N = 3, 38%), and ATC (N = 1, 12%). Median age was 58 years (range, 38-63 years). Seven were women (1 man:7 women) and the majority (N = 7, 88%) were White. All patients were Eastern Cooperative Oncology Group (ECOG) 0 to 1. Of the 4 patients with PDTC and ATC, 3 (75%) had PTC on prior histology (ie, presumed transformation). Seven had fusions in *NTRK3* (88%), and 1 in *NTRK1* (12%). Two patients’ tumors were *TP53*-mutated (25%) and 1 was *TERT* promoter–mutated (12%). All were stage IVC at diagnosis, with all 8 having metastases to the lungs (100%), 2 to the bones (25%), 2 to muscle (25%), 1 to the liver (12%), and 1 to the brain (12%; Table 1).

**Table 1.** Baseline characteristics, treatment details, and outcomes

Characteristic	ATC (N = 1)	PDTC (N = 3)	PTC (N = 4)	Total
Median age at diagnosis (range), y	61	44 (38-62)	59 (58-63)	58 (38-63)
Race	1 (100%) White	2 (67%) White, 1 (33%) Black	4 (100%) White	7 White (88%), 1 Black (12%)
Sex	1 (100%) male	3 (100%) female	4 (100%) female	7 female (88%), 1 male (12%)
*NTRK* fusion	*ETV6::NTRK3* exon 4::exon 14 chromosomes 12::15	*ETV6::NTRK3* exon 4::exon 13 chromosomes 12::15 *VIM::NTRK3* exon 8::exon 14 chromosomes 10::15 *ETV6::NTRK3* exon 4::exon 14 chromosomes 12::15 (developed *NTRK3* F617I)	*RBPMS::NTRK3* exon 5::exon 14 chromosomes 8::15 *ETV6::NTRK3* exon 5::exon 14 chromosomes 12::15 *EML4::NTRK3* exon2::exon14 chromosomes 2::15 *TPR::NTRK1* exon 22::intron 9 chromosome 1	7 *NTRK3* (88%), 1 *NTRK1* (12%)
*TP53*	0	1 mutated (33%)	1 mutated (25%)	2 mutated (25%)
*TERT*	0	1 mutated (33%)	0	1 mutated (12%)
Median TMB (range), μ/MB	1.7	1.7 (1.4-3.4)	2.6 (1.7-3.4), 2 missing	1.7 (1.4-3.4)
Mets sites	Lung	3 lung, 1 muscle	4 lung, 2 bone, 1 muscle, 1 brain	8 lung (100%), 2 to bones (25%), 2 muscle (25%), 1 liver (12%), 1 brain (12%)
Previous RAI	1 (100%) no	2 (67%) yes, 1 (33%) no	4 (100%) yes	6 yes (75%), 2 no (25%)
Previous radiation	1 (100%) yes	1 (33%) yes, 2 (67%) no	1 (25%) yes, 3 (75%) no	3 yes (38%), 5 no (62%)
Median time from diagnosis to systemic therapy start (range), mo	1.36	7.03 (6.36-10.7)	34.19 (9.83-106.16)	19.98 (1.36-106.16)
Previous systemic therapies	1 (100%) lenvatinib then sorafenib	1 (33%) none 1 (33%) sorafenib 1 (33%) lenvatinib	1 (25%) none 2 (50%) lenvatinib, then sorafenib 1 (25%) cabozantinib	
Treatment line of larotrectinib (range)	3	2 (1-2)	2.5 (1-3)	2 (1-3)
Response	1 (100%) PR	2 (67%) PR, 1 (33%) SD	1 (25% CR), 3 (75%) PR	1 CR (12%), 6 (75%) PR, 1 SD (12%)
Median DOR (range), mo	20.2	15.4 (6.1-20.3)	23.6 (22.3-47.1)	24.6 (6.1-47.1)
Median PFS (range), mo	23.33	17.5 (7.1-21.1)	34.6 (24.7-48.7)	24.7 (95% CI, 11.3-38.1)
Median OS (range), mo	23.33	30.1 (12.4-57.9)	56.8 (NE)	43.8 (29.8-56.8)
Alive at last follow-up?	1 no (100%)	1 yes (33%) 2 no (66%)	3 yes (75%) 1 no (25%)	4 yes (50%) 4 no (50%)

Abbreviations: ATC, anaplastic thyroid cancer; CR, complete response; DOR, duration of response; Mets, metastases; OS, overall survival; PDTC, poorly differentiated thyroid cancer; PFS, progression-free survival; PR, partial response; PTC, papillary thyroid cancer; RAI, radioactive iodine.

**Figure 1.**
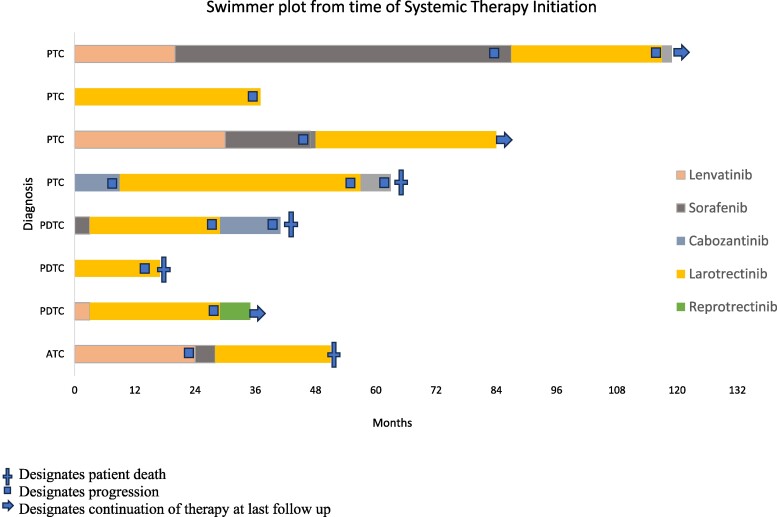
Swimmer plot showing treatment information for study cohort. A blue cross designates a patient death. An arrow designates continued treatment at last follow-up. A blue square indicates disease progression.

In terms of prior therapy, all 8 patients underwent thyroidectomy. Three patients underwent radiation to the thyroid bed (38%). Six patients (75%) received systemic therapy prior to larotrectinib: 4 with lenvatinib (50%), 3 with sorafenib (38%), and 3 with cabozantinib (38%; Fig. 1). Larotrectinib was given as a median second line of therapy (range: 1-4).

On larotrectinib, 1 patient achieved CR (12%), 6 patients (75%) PR, and 1 had stable disease (12%), with an ORR of 88%. Treatment-related adverse events (per CTCAE v5.0) were observed in 4 patients (50%), with 2 patients exhibiting grade 1 fatigue (25%), 1 patient exhibiting grade 1 myalgias (12%), and 1 patient having grade 1 peripheral edema (25%). No patients required dose reduction or medication discontinuation due to treatment-related adverse events.

**Figure 2.**
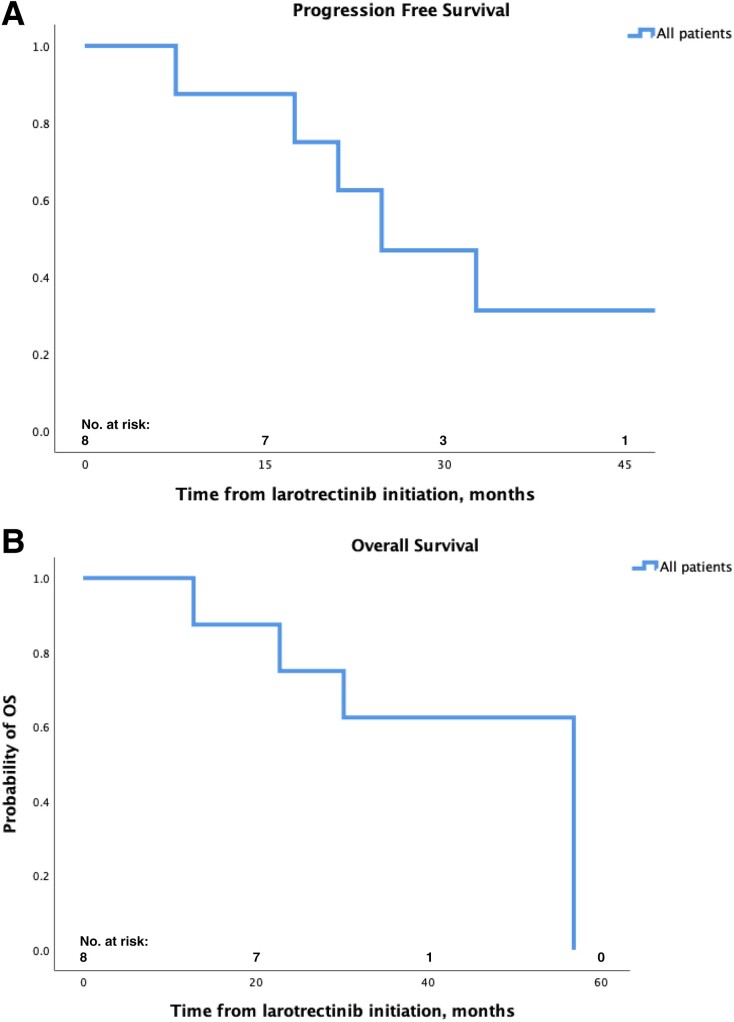
Kaplan-Meier curve of A) progression-free survival and B) overall survival for patients receiving larotrectinib within the study cohort.

Six patients (75%) experienced disease progression as of last follow-up. Median follow-up was 51.8 months. mPFS for all patients was 24.7 months (95% CI, 11.3-38.1 months) (Fig. 2A). Patients with PTC had longer mPFS (34.6 months [95% CI, 24.7-48.7 months]) than those with PDTC (17.5 months [7.1-21.1 months]; *P* = .017); the single patient with ATC had a PFS of 23.3 months. DOR was longer for patients with PTC (median 31.6 months [22.3-47.1 months]) compared to those with PDTC (median 15.4 [6.1-20.3 months]; *P* = .009); the single patient with ATC had a DOR of 20.2 months.

Four patients (50%) have died as of last follow-up. mOS was 43.8 months [29.8-56.8 months] (Fig. 2B). Patients with PTC had numerically longer OS (56.8 months [not evaluable]) compared to patients with PDTC (30.1 months [2.4-57.9 months]; *P* = .071) and the patient with ATC (30 months [not evaluable]; *P* = .046).

Four patients (50%) went on to receive therapy after larotrectinib: 1 with repotrectinib (12%), 1 with lenvatinib (12%), and 2 with cabozantinib (25%). One patient, on progression on larotrectinib, was found to have an *NTRK3* F617I fusion, an acquired resistance mutation, and was initiated on repotrectinib, achieving a PR. She has sustained this response for 9 months as of last follow up.

## Discussion

In this largest observational series to date of *NTRK*-positive thyroid cancer patients treated with *NTRK* inhibitor therapy, 8 patients with metastatic thyroid cancer harboring *NTRK* fusions achieved sustained responses with the *NTRK* inhibitor larotrectinib, with an mPFS of nearly 25 months and an mOS of 43 months. Patients with PTC had the longest PFS and OS, consistent with the natural history of the disease. Larotrectinib administration was safe and tolerable, with no patients needing a dose reduction or discontinuation due to toxicity.

Seven of our patients were diagnosed with fusions of the *NTRK3* gene, which encodes for a single-pass transmembrane receptor tyrosine kinase, the NT-3 growth factor receptor (TRKC), and one patient had a fusion in *NTRK1*. Together with *NTRK2* and *3*, the protein products of the 3 *NTRK* genes regulate the development, maintenance, and function of neural tissues [22]. Somatic intrachromosomal or interchromosomal rearrangements involving *NTRK1*, *NTRK2*, or *NTRK3* may be found as oncogenic drivers in a wide range of tumor types. In almost all such cases, the amino-terminal portion of a partner gene product is fused to the carboxyl-terminal portion (inclusive of the tyrosine kinase domain) of the *NTRK* gene product, leading to a constitutively activated kinase. Interestingly, one of our patients had an unusual *NTRK3* fusion with *VIM* on chromosome 10p3, a rare alteration observed only in thyroid cancers that has not been well characterized in the literature [23]. Despite this alteration, the patient had a robust DOR of 15.4 months with a PFS of 17.5 months and an OS of 30.1 months.

In the most recent combined analysis of phase 1/2 basket trials of larotrectinib including 24 DTCs and 7 ATCs with NTRK fusions, the ORR was 65% with 3 CRs and PRs; ORR was 79% in DTC and 14% in ATC [10]. Similarly, our cohort demonstrated favorable response rates and survival with *NTRK*-targeted therapy, with an mPFS of 34.6 months and OS of 56.8 months in patients with PTC, and the majority of patients still alive at time of last follow-up. PDTC patients in our cohort demonstrated an mPFS of 17.5 months and an mOS of 30.1 months, which is a significant improvement over the historic PFS observed for metastatic PDTC [24]. Our patient with ATC survived 23.33 months after larotrectinib initiation, which is more than 4 times the expected life expectancy of patients with ATC [25]. Our cohort's ORR of 88% exceeded that of the Cabanillas basket study (65%) [10] or of the entrectinib integrated study (57%) [4, 12, 13], while our mPFS of 34.6 months was lower than the 44.0 reported by Cabanillas et al [10].

On progression on larotrectinib, one patient was found to have an *NTRK3* F617I mutation, a rare, acquired resistance mutation in the gatekeeper region of the *NTRK3* gene (as opposed to the more commonly involved solvent front). Interestingly, in one study of 18 patients treated with a first-generation *NTRK* inhibition who developed resistance mutations, only one developed an *NTRK* F1617I mutation [26]. Similar to prior reports, treatment with repotrectinib was successful in overcoming resistance to earlier-generation NTRK inhibitors in the patient with PDTC in our study, who has maintained a response for more than 9 months as of May 2024 [27].

The major limitations of this analysis include its small size, given the relative rarity of *NTRK* fusions in adult patients with thyroid cancer, and heterogeneity in the cohort in terms of histology and prior treatment. As this was a retrospective, chart review-based study, details on adverse events and tolerability were limited. Finally, since larotrectinib was preferentially used at our institution, we are not able to comment on efficacy or tolerability with entrectinib.

In summary, in this retrospective, single-institution cohort of patients with *NTRK* fusion-positive thyroid cancer, *NTRK* inhibition led to deep and durable responses, with survival surpassing historic benchmarks for ATC and PDTC. We also demonstrated a 35-month mPFS and 57-month mOS among metastatic PTC patients, with the majority living at time of last follow-up. This study underscores the importance of molecular testing for patients with recurrent/metastatic thyroid cancer to identify actionable alterations including *NTRK*, as well as more common driver alterations including *BRAF* and *RET*, and enable selection of the most efficacious systemic therapy.

## Data Availability

Some or all data sets generated during and/or analyzed during this study are not publicly available but are available from the corresponding author on reasonable request.

## Abbreviations

ATC: anaplastic thyroid cancer
CR: complete response
DOR: duration of response
mOS: median overall survival
mPFS: median progression-free survival
NGS: next-generation sequencing
*NTRK*: neurotrophic tyrosine receptor kinase
ORR: overall response rate
OS: overall survival
PDTC: poorly differentiated thyroid cancer
PFS: progression-free survival
PR: partial response
PTC: papillary thyroid cancer
RAI: radioactive iodine
